# A situational analysis of the mental health system of the West Region of Cameroon using the World Health Organization’s assessment instrument for mental health systems (WHO-AIMS)

**DOI:** 10.1186/s13033-022-00528-9

**Published:** 2022-03-12

**Authors:** Michael guy Toguem, Manasi Kumar, David Ndetei, Francois Erero Njiengwe, Frederick Owiti

**Affiliations:** 1 Department of Psychiatry, University of Nairobi, Nairobi, Kenya; 2 Department of Social Sciences, University of Douala, Douala, Cameroon

**Keywords:** Cameroon, Mental health system, WHO-AIMS, West Region

## Abstract

**Background:**

The publication of the *World Health Report of 2001* by the World Health Organization (WHO) raised awareness of mental health worldwide. It reported a higher burden of mental illnesses in developing countries, with fewer resources to address the conditions. Since then, many initiatives have been taken in Cameroon to improve the mental health of the population, but these efforts lack local scientific evidence to support them. This study aims to describe the mental health services available in the West Region of Cameroon and to provide evidence-based support to this process.

**Method:**

We used the World Health Organization Assessment Instrument for Mental Health systems (WHO-AIMS) version 2.2 to collect, analyze, and report the data on mental health services offered in 2020 in the West Region of Cameroon. We extracted our data from the registers of 10 mental health facilities of the region, and we interviewed the staff in these facilities and at the Ministry of Public Health.

**Results:**

The region is divided into 20 health districts, of which six offered mental health services. As a whole, Cameroon had a mental health policy, mental health plan, but no mental health legislation or emergency plan. There was no specific budget for mental health in the country. In the West Region there were not any psychiatrists. Mental health services were provided by nurses, psychologists, general practitioners, and neurologists. There were 1.87 human resources in mental health facilities per 100,000 population, of which 1.4 were nurses. 1 in 1.4 of these nurses worked in the main city. There was no formal link of mental health services with other sectors and no publication in the previous five years about mental health in the region referenced on PubMed.

**Conclusion:**

In 2020, the access to mental health services in the West Region of Cameroon was inequitable. The approach to mental health system development was not inclusive and not supported by enough scientific evidence.

## Introduction

In 2001, the World Health Organization (WHO), in their *World Health Report*, focusing on mental health, identified a tremendous treatment gap and suggested remedial measures. This report stated that mental and behavioral disorders affected more than one-quarter of the world’s population in their lifetime and that this burden of mental ill-health was expected to grow. This burden of mental health was higher in Lower- and Middle-income Countries (LMIC) than Higher-income Countries (HIC). LIMC were also found to be the one with fewer resources to address this burden. Following this report, the World Health Organization Assessment Instrument for Mental Health Systems (WHO-AIMS) was built for the initial assessment and monitoring of mental health systems. [1, 2].

Since the release of this report, HIC have further managed to narrow their mental health gap [3]. In LMIC, making use of the WHO-AIMS, many countries have managed to improve their mental health systems through improved data capturing and setting up of national plans and policies. However, much work still remains to be done. Brazil has been able to improve the enforcement of the human rights of individuals with mental illnesses, stimulate a mental health research community in the country, reduce the number of custodial psychiatric beds by transferring these beds to primary healthcare mental health services, and provide some specialized mental health services for children and adolescents [4, 5]. The WHO South-East Asia region represents one-quarter of the world’s population. Seven out of the 11 countries in this region have used the WHO-AIMS to build baseline information to improve their mental health systems [6].

In the African setting, the WHO, through Gureje and Alem, appealed to African governments to invest in mental health programming and service scale-up by updating their policies, educating the population about the value of sound mental health, providing mental health services, and promoting mental-health-related research. After this appeal, several initiatives were taken up in Kenya, including many initial assessments using the WHO-AIMS [7, 8], the development of a mental health policy, and so on [9]. In countries such as Uganda, Ethiopia, South Africa, Ghana, and Nigeria, initial national assessments of the mental health systems were carried out using the WHO-AIMS [10–14].

Cameroon is a lower-middle-income country in Central Africa, the eastern neighbor of Nigeria. Cameroon had a population of approximately 25 million people and a life expectancy at birth of 59 years in 2019 [15], and the country has English and French as its national languages [16]. The sex ratio of Cameroon is 1:1. The urban population was 57.56 per cent of the total population in 2020. The country is divided into 10 administrative units called regions [17, 18]. The national health system plan follows the same regional division [19].

Like many other LMIC, Cameroon lags in terms of mental health infrastructure. In 2017, mental health disorders accounted for 2366.29 disability-adjusted life years per 100,000 population in Cameroon [20]. Following the recommendations of the WHO, mental health was introduced in the minimum package of healthcare services in Cameroon [19]. The first mental health plan and policy were published in 2016 [21, 22]. The country’s psychiatrists are mostly concentrated in the capital cities: Douala and Yaounde, which are located in the Littoral and Central Regions. This leaves relatively remote areas such as the West Region with fewer resources. It also raises concerns about how Cameroonians with mental illnesses in the West Region of Cameroon find support and care and what sort of mechanisms are in place for the promotion, restoration, and maintenance of their mental health.

By undertaking this study, we aim to understand the spectrum of mental health disorders and the available resources, which will inform the planning of mental health services and provide a basis for monitoring the progress in mental healthcare services in light of a devolved healthcare system in the West Region of Cameroon.

## Method

The WHO-AIMS version 2.2 was used to collect, analyze, and report data on mental health systems and services of the West Region of Cameroon. The data was collected from 1 to 14 February 2021, based on the year 2020.

The focus of the *World Health Report 2001* was to underscore the importance of mental health. The recommendations were summarized in 10 key points, targeting the need to strengthen mental health systems. To be able to make an initial assessment and a follow-up monitoring on these dimensions, the WHO-AIMS was proposed as a monitoring tool for countries to use. The WHO-AIMS is a questionnaire that provides key information about mental health systems. It is divided into six domains, 28 facets, and 156 items. The six domains are interdependent, conceptually interlinked, and somewhat overlapping. The domains are:

- Domain 1: Policy and legislative framework
- Domain 2: Mental health services
- Domain 3: Mental health in primary care
- Domain 4: Human resources
- Domain 5: Public education and links with other sectors
- Domain 6: Monitoring and research [1]

Usually, this tool is populated by government ministries of health where the departments/divisions of mental health belong. In many countries, such as Cameroon, there is a paucity of resources. Documenting the available resources and mapping existing services would spur forward-going action and progress in research-based advocacy for strengthening mental health systems in Cameroon.

For the purpose of our study, the questions of the WHO-AIMS were converted into a simpler format to make data collection more convenient, as this exercise was carried out by a post-graduate psychiatry resident as a pilot initiative. For example, item 2.2.3: gender distribution of users treated through mental health outpatient facilities was reformulated into the following two questions:

- Number of female users treated through your mental health outpatient facility in the year 2020.
- Number of users treated through your mental health outpatient facility in the year 2020.

After the reformulation, shorter questionnaires seeking specific information from each institutional type or document where the data would be collected were developed. This included the following:

- Mental hospitals, forensic in-patient units, day treatment facilities, mental health outpatient facilities, community residential facilities, community-based psychiatric inpatient units, and other residential facilities. The sources of information were the mental health workers in charge of patients’ care and the records of the facilities.
- Ministry of Public Health, Office for Mental Health. Here the source of information was one of the people in-charge of national mental health.
- West regional delegation for the Ministry of Public Health, Cameroon. Here, the sources of information were the two heads of human resources.
- The penal and civil code of Cameroon.
- The national health development plan of Cameroon 2016–2020.
- The national policy for mental health of Cameroon.
- The national mental health plan of Cameroon.
- The harmonized programs for medical schools in Cameroon (programmes harmonisés de la filière médicale au Cameroun).

The questionnaires looked at the 28 facets. We received authorization for the research from the West regional delegation of the Ministry of Public Health. They also provided us with a list of the public hospitals in the region. We approached the Association du Personnel de Sante mentale de l’Ouest (APE SANO), which is the network of mental-health professionals of the West Region of Cameroon. The association provided us with a list of mental-health professionals of the West Region of Cameroon with their respective contact information and place of work. We obtained research approvals from the directors of all the identified health facilities. After this, we excluded from the study the facilities that did not offer mental health services. The data was collected through a phone call to the informant at the Office of Mental Health at the Ministry of Public Health (20 min), a face-to-face interview at the West regional delegation for the Ministry of Public Health Cameroon (30 min), face-to-face interviews (10 min), exploration of records at the mental health facilities, and finally, the exploration of national documents. The data was analyzed using Microsoft Excel 2013. This study was undertaken as a research project for the degree of master of medicine in psychiatry by the first author. We received ethical clearance Number 2490 IEC-UD/10/2020/T from the ethical committee of the University of Douala, and KNH-ERC/RR/842 from the ethical committee of the University of Nairobi/Kenyatta national hospital.

## Results

The study was conducted in the West Region of Cameroon. In 2015, it had an estimated population of 1,921,590 people, divided into 20 health districts, spread on an area of approximately 13,872 square kilometers. People aged 15 and below represented 44.71 percent of the population [23, 24]. The most used language in the region is French, and the main ethnic groups are Bamileke and Bamoun. Religious groups include Muslims, Animists, Catholics, and other Christian confessions. The population is a mix of rural and urban communities.

### Policy and legislative framework

A mental health policy is an official statement by a government or health authority that provides the overall direction for mental health by defining a vision, value, principles, and objectives and establishing a broad model for action to achieve that vision [25]. Cameroon’s mental health policy was last revised in 2016 and includes the following components: (1) developing a mental health component in primary healthcare, (2) human resources, (3) involvement of users and families, (4) advocacy and promotion, (5) human rights protection of users, (6) equity and access to mental health services across different groups, (7) quality improvement, (8) monitoring system, (9) developing community mental health services, and (10) financing. It does not include the downsizing of large mental hospitals. The National Essential Medicines list includes all the categories of psychotropics: (1) antipsychotics, (2) anxiolytics, (3) antidepressants, (4) mood stabilizers, and (5) antiepileptic drugs.

A mental health plan details the strategies and activities that will be implemented to realize the vision and achieve the objectives of a mental health policy [25]. Cameroon’s last revision of the mental health plan took place in 2016 and included: (1) human resources, (2) advocacy and promotion, (3) human rights protection of users, (4) financing, and (5) monitoring system. This mental health plan does not include the development of community mental health services, the downsizing of large mental hospitals, any reformation of mental health hospitals to provide comprehensive care, equity of access to mental health services across different groups, and quality improvement. Even though users and families are mentioned in this plan, it is not clear what role they will play. A timeframe and specific goals are mentioned in the plans, some of which have been fulfilled in the last year, but there is no specific budget dedicated to mental health. There is no disaster/emergency preparedness mental health or psychosocial plan, and there is no current mental health legislation even though there are some considerations about mental health in the penal, civic, and family code. For example, in Chapter II, Articles 78 and 79 of the penal code, it is said that a person with a mental illness, including involuntary intoxication, can have his penal responsibility canceled or attenuated. In Chapter II, Article 489 of the civil code talking about the age of majority, it is said that the adult who is in a habitual state of imbecility, dementia, or fury, should be prohibited from the majority status, even when this state presents lucid intervals.

The heads of mental health units reported that during the year 2020, there was no inspection or training of mental-healthcare workers on human rights. Mental health was financed through the general budget allocated for health. All the mental health hospitals of the region are privately owned, and do not receive money from the government. There is no social assurance scheme, and there is no free access to essential psychotropic medicines. One-day treatment of antipsychotic medication is 8.7 percent, and antidepressant medication is 20.4 percent of the minimum daily wage. These calculations are based on the current minimum monthly wage of 36,270 FCFA (Franc de la communauté financière africaine) and the cost of the cheapest antidepressant and antipsychotic as listed on the application Med Index where drug stores of the region list their available drugs with the prices. Worker’s insurance benefits whereby workers pay a premium to private insurers in order to have partial or total coverage of their health bills exist, but the number of people covered is unknown.

### Mental health services

There is a National Mental Health Authority led by the vice-director of mental health affiliated with the Ministry of Public Health. The vice-director provides (1) advice to the government on mental health policy and legislation, (2) is involved in service, (3) and is incharge of service management. There is no monitoring or quality assessment of these mental health services that are currently performed. In the West Region, all community-based psychiatric inpatient units are affiliated to a mental health outpatient facility in the same hospital. Both are run by the same personnel except in the referral hospital of the region, where only the head of the neuropsychiatric unit worked both in the outpatient and inpatient units. Mental health services are organized in terms of service areas known as districts. There are 20 districts in the region, but only six of them have a mental health facility. These six districts are Batcham, Foumban, Mbouda, Dschang, Mifi, and Bangangte. The other 12 facilities often refer their mentally unwell patients to the closest mental health service available.

In the region, we counted eight outpatient mental health facilities and two neuropsychiatric units that offer outpatient services. The neuropsychiatric unit is a combination of a psychiatric and a neurological unit. A neurologist who diagnoses and treats psychiatric patients leads this service. For this research, we will consider the neuropsychiatric unit as offering core psychiatric services given this dual role it serves. All these facilities are organizationally integrated with mental health outpatient facilities. There is no mental health service dedicated to children and adolescents in the region. All the psychiatric diagnoses and prescriptions in the region are made by mental health nurses and neurologists. According to the registers, these facilities treated 127 users per 100,000 members of the general population (2,448 users) in the year 2020. Of all users treated in mental health outpatient facilities, 51 percent were female, and 19 percent were below 18 years old.

In 2020, the users treated in outpatient facilities were diagnosed with mental and behavioral disorders such as due to psychoactive substances (4 percent); schizophrenia, schizotypal, and delusional disorder (14 percent); mood disorders (22 percent); neurotic, stress-related, and somatoform disorders (14 percent); disorders of adult personality and behavior (1 percent); and other diagnoses (epilepsy, organic mental disorders, mental retardation, behavioral and emotional disorder with onset usually occurring in childhood and adolescence, disorders of psychological development), which represented 37 percent (see Table 1). The category of other diagnoses was dominated by epilepsy. When this information was collected, it was found that there was some missing information in the registers, so we have to keep this limitation in mind. We observed some unique diagnoses such as “schizophrenie induite par l’usage de substance psychoactive” (substance-induced schizophrenia), “psychose maniaco depressive” (manic depressive psychosis), “psychose hallucinatoire chronique” (chronic hallucinatory psychosis), and so on. The average number of contacts per user was two. Half of the outpatient facilities provided follow-up care in the community, while 10 percent (01) had mobile mental health teams. There is no day treatment facility. In terms of available treatments, 20 percent of the outpatient facilities offered psychosocial treatments. Clinical psychologists delivered these psychosocial treatments. 42.9 percent of mental health outpatient facilities had at last one psychotropic medicine of each therapeutic class (antipsychotic, antidepressant, mood stabilizer, anxiolytic, and antiepileptic medicines) available in the facility, and all of the facilities had access to them in the facility or a near-by pharmacy all year round.

**Table 1:** Users treated in outpatient mental health facilities by diagnosis (%)

	Mental hospitals	Community-based psychiatric units	Mental health facilities
Mental and behavioral disorders due to psychoactive substance use (F10–F19)	38 (24%)	62 (3%)	100 (4%)
Schizophrenia, schizotypal, and delusional disorders (F20–F29)	61 (38%)	291 (13%)	352 (14%)
Mood (affective) disorders (F30–F39)	14 (9%)	517 (23%)	531 (22%)
Neurotic, stress-related, and somatoform disorders (F40–F48)	8 (5%)	338 (15%)	346 (14%)
Disorders of adult personality and behavior (F60–F69)	1 (0.6%)	19 (1%)	20 (1%)
Others	11 (7%)	893 (39%)	904 (37%)
Users treated in outpatient mental health facilities	160	2288	2448

There were seven community-based psychiatric inpatient units (including the two community-based neuropsychiatric inpatient units) in the region for a total of 0.62 exclusive psychiatric beds per 100,000 population (see Table 2). Based on the estimations of mental health nurses, one unit had 20 percent, and another one did not admit patients aged below 18 years old. One-third of community-based psychiatric inpatient units have at least one psychotropic medicine of each therapeutic class (antipsychotic, antidepressant, mood stabilizer, anxiolytic, and antiepileptic medicines) available at the facility.

**Table 2:** Mental-health workers and beds (per 100,000 population)

Health workers	Mental hospitals (per 100,000 population)	Community-based psychiatric units (per 100,000 population)	Mental-health facilities (per 100,000 population)
Psychiatrists	0 (0)	0 (0)	0 (0)
Other medical doctors, not specialized in psychiatry	0 (0)	3 (0.16)	3 (0.16)
Nurses	10 (0.52)	17 (0.88)	27 (1.41)
Psychologists	0 (0)	2 (0.10)	2 (0.10)
Social workers	0 (0)	0 (0)	0 (0.00)
Occupational therapists	0 (0)	0 (0)	0 (0.00)
Other health or mental health workers	4 (0.21)	0 (0)	4 (0.21)
Totals	14 (0.73)	22 (1.14)	36 (1.87)
Beds	30 (1.56)	12 (0.62)	42 (2.19)

There were two mental hospitals in the region with 1.6 beds per 100,000 population (see Table 2). Of the users of mental hospitals, 40.6 percent were female. The diagnoses among all the users of mental hospitals were as follows: mental and behavioral disorders due to psychoactive substance use (24 percent); schizophrenia, schizotypal, and delusional disorders (38 percent); mood disorders (9 percent); neurotic, stress-related, and somatoform disorders (5 percent); disorders of adult personality and behavior (0.6 percent), and others (7 percent) (Table 1). The number of users of mental hospitals was 8.3 per 100,000 population (160 users). All mental hospitals had at least one psychotropic medicine of each therapeutic class (antipsychotic, antidepressant, mood stabilizer, anxiolytic, and antiepileptic medicines) available in the facility. There was no forensic or residential facility in the region.

The status of hospital admission to mental health institutions regarding whether it was voluntary or involuntary was not recorded. Nurses reported that 19 percent of patients in community-based psychiatric inpatient units and 30 percent of the inpatients in mental hospitals were restrained or secluded at least once.

The ratio of beds located in Bafoussam, which is the largest city in the region, compared to those available in the whole region, was three. From the perspective of the heads of mental health units, users who were linguistic, ethnic, or religious minorities did not have any issue with access to mental health services.

### Mental health in primary health care

In terms of physician-based primary healthcare clinics offering mental health services, the heads of mental health units reported that only a few (14.3 percent) had assessment and treatment protocols for key mental health conditions available: 50 percent of full-time primary healthcare doctors make at least one referral per month to a mental health professional as a follow up on their transferred patients; 29 percent of the primary healthcare doctors in primary healthcare facilities interacted at least once a month with a mental health professional. 14 percent of the primary healthcare facilities and none of the mental hospitals interacted with a complementary/alternative/traditional practitioner.

Non-doctor and non-nurse primary healthcare workers were not allowed to prescribe psychotropic medications under any circumstances. Primary healthcare doctors and mental health nurses were allowed to prescribe psychotropic medications without restrictions. However, on the ground, this is done by psychiatric nurses and neurologists. As for the availability of psychotropic medicines, almost all physician-based primary health care clinics had at least one psychotropic medicine of each therapeutic category (antipsychotic, antidepressant, mood stabilizer, anxiolytic, and antiepileptic) available.

### Human resources

Based on the harmonized program in Medical Education Training at the School of Medicine in Cameroon, in the curriculum, of the total training hours for medical doctors, 0.9 percent is devoted to mental health, and this training is mainly theory-based. Nurses also receive training on mental health, but we could not get more information on this. In terms of refresher training, two nurses, belonging to a faith-based primary healthcare facility, had received at least two days of refresher training in mental health, while none of the primary healthcare doctors received such continuous professional development training.

As per the heads of human resources at the West regional delegation for the Ministry of Public Health, the whole region had one school that trained mental health nurses. The school is in its second year of existence, and the course they offer is of three-year duration. There is only one school of medicine and several schools of nursing, and these other schools do not train mental health professionals. Among the mental healthcare staff, we counted one nurse who attended refresher training on the rational use of drugs while none attended refresher training on psychosocial interventions or child/adolescent mental health issues. They said that there are no consumer or family associations for people with mental illness in the region.

There are 1.87 human resources personnel working in mental health facilities per 100,000 population in the region. The breakdown according to the profession is as follows: zero psychiatrists, 0.1 neurologists, 0.05 general practitioners; more details in Table 2. 52 percent of psychologists and nurses work only for government-administered mental health facilities, 45 percent only for mental health non-governmental organizations, for-profit mental health facilities, or private practice, and 3 percent work for both. One nurse in 1.4 works in the largest city: Bafoussam.

Regarding the workplace, as for the other medical doctors (that is, those not specialized in mental health), two neurologists work in mental health outpatient facilities and the community-based psychiatric inpatient units of the same hospitals, one general practitioner works in a community-based psychiatric inpatient unit and none in mental hospitals. 17 nurses worked in mental health outpatient facilities, 16 in community-based psychiatric inpatient units, and 10 in mental hospitals. Two psychologists worked in hospitals that included an outpatient facility and a community-based psychiatric inpatient unit; each covered both units. None of them worked in a mental hospital. Finally, regarding the other health- or mental-health workers, four worked in mental hospitals, while none worked at the community-based facilities because, in these facilities, they belong to other units and assist in case they are needed.

There were 1.4 nurses per bed assigned specifically for psychiatric patients in community-based psychiatric inpatient units, compared to 0.33 per bed in mental hospitals. Other mental healthcare staff (for example, psychologists, social workers, occupational therapists, other health- or mental-health workers) were 0.13 per bed in mental hospitals.

### Public education and links with other sectors

There is a foundation in the region, Fondation Olympia Jujitsu Cameroun (FOJCAM), that oversees public education and awareness campaigns on psychoactive substance use in the entire country. These campaigns have targeted the general population, adolescents, and women. There have not been any public educational awareness campaigns targeting professional groups.

As per the informant at the Ministry of Public Health, there is no legislative or financial support for employment, no provision against discrimination at work, or discrimination in housing for people with mental disorders. There is no formal collaborative program with other health- and non-health agencies nationally.

The mental hospitals do not provide livelihood or life-skill opportunities to individuals with severe mental disorders. None of the mental-health workers in the region worked for a school. Only one nurse provided mental health services at one of the prisons of the region, and he sees at least one prisoner per month from the prison of his district and has over a dozen districts under him. The mental-health workers of the region are not mandated or involved in mental health promotion activities targetting police officers, judges, and lawyers. Finally, there is no social welfare benefit for disability in the region.

### Monitoring and research

A formally defined list of individual data items that ought to be collected by all mental health facilities exists and includes the number of inpatient admissions, number of days spent in hospital, diagnosis, and number of users treated even though none of these facilities collected data on the number of days spent in admission as seen in the registers. This information is sent every three months to the West Regional delegation for the Ministry of Public Health. The government does not produce any report on mental health services in the region. None of the mental health professionals of the region are involved in research. On PubMed, there has been no publication on mental health in the region in the past five years. Meanwhile, there are 40 health-related publications from the region identified on PubMed.

## Discussion

Despite the paucity in actual services, monitoring, and reporting of mental health of its population, in recent years in Cameroon, there has been some marked improvement in the mental health system through proactive government engagement in this area. This is evidenced by the introduction of mental health curriculum in the national harmonized program for medical schools since 2015 [26]. In 2016, there was the introduction of mental health services in the essential health package, which provides the necessary bare-bones services [26], and this was followed by the development of a mental health policy, the shaping of a mental health plan, and the presence of all classes of psychotropics on the essential medicine list to be made available at public facilities in the country [19, 21, 22]. With the Coronavirus pandemic, the government further improved the country’s mental health services and hired more mental health workers [27]. In the West Region of Cameroon, these efforts translated into services being integrated into six districts out of the 20 now offering mental health services. There was a psychiatric nursing school in its second year of operation at the beginning of 2020. Despite these noticeable advances, there is still a tremendous amount of work left for mental health in the region and the country.

### Access to care

Currently, in the West Region of Cameroon, mental health services are not available in 14 out of the 20 health districts. In the six health districts where they are available, there are three times more beds per patient in the largest city, Bafoussam, and one nurse for every 1.4 work there. One-day treatment with antipsychotic and antidepressant medication costs 8.7 percent and 20.4 percent of the minimum daily wage, respectively.

The current cost of antipsychotic and antidepressant medication seems better than the 37 percent and 7 percent of the minimum wage observed in Uganda [28]. It does not serve all Cameroonians equitably. There are many financial barriers to access to mental healthcare in the region. These include the lack of social welfare benefits for disability, the absence of a social assurance scheme, no free access to essential psychotropic medicines, and no legal provision for mentally disabled people to have access to jobs. It places a heavy burden on patients with mental illnesses and their families. The districts with no mental health services should have a referral system with those that provide access to mental health services, and then they should develop their own rudimentary service structure. There should be legal provisions to facilitate access to jobs for people with mental disabilities and social welfare for the most disabled mentally-ill people. The government could consider building a regional or national insurance scheme to protect the population from catastrophic financial risk related to mental illnesses [2].

### Mental health awareness

In the West Region, FOJCAM was the only one offering public education on mental health; however, they only focused on substance use. Apart from this, there is no association serving mentally unwell individuals or their families. Without such social- and advocacy-based protection, stigma and discrimination against mental illness are a perpetual issue [21, 29].

The mental health of the population is an important consideration in the legislation of Cameroon. However, this not enough. There is a need for specific policies to protect the rights of people with mental illness by protecting them against discrimination, providing motivation to employers to hire them, and making the working environment mentally healthy. These specific policies, can constitute a mental health legislation, and should be guided by the United Nations General Assembly Resolution 46/119 on the Protection of Persons with Mental illnesses, of which Cameroon is a member state. It will prevent people with mental illnesses from abuse and seek to improve their health. There is also a need for an alcohol and drug policy to prevent substance-use disorders and refer these individuals to appropriate rehabilitation and detoxification including associated mental health services [2, 30]. The members of the Office of Mental Health at the Ministry of Health should be the stewards of the mental health awareness process, and offer guidance on treatment, prevention, and promotional activities. In partnering with associations such as FOJCAM, efforts can be made to plan a mental health awareness strategy and include it in the mental health plan because the ministry’s active involvement might lead to a better outcome, as suggested by the Mental Health Leadership and Advocacy Program in West Africa [31]. The awareness activities should be extended to include suicide prevention, depression, psychosis, and any other identified priority. They should also target different professional groups such as medical associations, legal, educational, or private-sector associations [2].

At the community level, people with mental illness and their families need to be encouraged to form associations and actively promote their rights and well-being. Famous figures such as musicians or athletes can also play a key role in shifting the public perception of mental health through different ways, including mass media such as Facebook, television, and YouTube. Reducing the stigma might improve the contact rate of 2 per patient observed in the region, which is very small compared to the 4.99 observed in Ghana [2, 32]. Improving the rights of people with mental illness might also reduce the seclusion and physical restriction rate of 19 percent, which is higher than the 10 percent reported in Ghana and lower than the 33 percent reported on average in LMIC [32, 33].

### Inclusion

The development of the current mental health policy and plan only involved professionals from the public health field. This is why it fails to consider the perspectives and inputs from the private sector, the financing system, the justice system, the education system, religious healers, traditional healers, traditional leaders, the people living with mental illness, and their families. The transdisciplinary nature of mental health leads to insufficiencies that can be noticed in those documents. It does not address the development of community mental health services, quality of care, and equity of access to care. This translates into very poor linkages with other sectors that we noticed in the region. This issue can only be addressed by bringing to the table all the stakeholders during the next revision of these documents and making efforts to gather a multidisciplinary team of mental health service providers [2].

### Community health

In the region, half of the outpatient facilities provided follow-up care in the community, while 10 percent (01) had mental health mobile teams focusing on reaching out to people with mental illness in the community. Currently, community mental health service is being offered as a unique initiative of the mental health worker. This should be done systematically, organized at the regional or national level by including it in the mental health plan and policy. It should also include preventive measures against suicide, gender-based violence, child abuse, among others, and promote mental wellness. This is particularly important because we found mood disorders to be the most common diagnostic category in the mental health facility, representing 22 percent of the diagnoses, and mood disorders are often preventable at a lower cost [2]. Mental health promotion should be aligned with the needs of each population group. They can include yoga, mindfulness, or meditation, which were found to reduce stress, improve mood, sleep, and school performance among children in the USA [34]. These activities can be used in collaboration with the other community health-related activities active in the region, such as vaccination, to make the process easier and more efficient.

### Evidence-based mental health system

None of the mental health workers of the region were involved in the research. There is no mental-health-related article about the West Region of Cameroon found on PubMed in the past five years. The heads of mental health services in all the facilities we visited shared that they report hospital-based mental health indicators every three months to the West regional delegation for the Ministry of Public Health, Cameroon. Despite this, there has never been a published mental health report by the delegation or the government on the state of affairs around mental health. All this translates into a lack of evidence to support the decisions made by the government about the mental health system in the region, and this gap is sorely felt at the national level, too [21, 22]. This weakness reflects at all levels in the current mental health system. First, the real burden of mental and behavioral disorders, mental health needs, specific diagnosis, their prevalence, and the factors contributing to their occurrence in the region are unknown. Since this information is not available, it is difficult to clearly define the position of mental health in terms of investment priority, the range of services needed for the population, the resources needed to provide these services, and, more specifically, the budget allocated to mental health and the number and quality of human resources needed. These might be some of the reasons why they are not mentioned in the current mental health plan. This lack of evidence compromises the quality of the entire process. The evidence-based approach is at the heart of efficacy and efficiency, and hence the government needs to put forward research as a central element in solving mental health problems [2]. To achieve this, the government will need to address several issues.

The government needs to improve the mental health workforce. Only 0.9 percent of the training of medical doctors is devoted to mental health in Cameroon. This is very small compared to the 10 percent observed in Uganda and the average 3 percent in LMIC overall [28, 33]. In addition, this training in Cameroon does not include any practical exposure to mental health. This poor consideration for mental health in the harmonized program for schools of medicine might be because this document was built before the introduction of mental health in the minimum package for health and the development of the first mental health policy and plan of Cameroon. The inclusion of clinical training and increasing the number of hours devoted to mental health might generate more vocations with regards to mental health and make doctors more capable of managing mental health problems. The mental health research capacity can be improved by partnering with skillful mental health researchers from other parts of the country and international partners to support the West Region in research capacity development. This can be achieved by training, involvement, and building career plans for mental health workers in the region that takes into account mental health related research. Mental health workers could team up with qualified global or regional mental health researchers in the context of research projects that will help to generate the evidence needed to build a stronger mental health system [2]. Support from United Nations agencies that aid mental health and psychosocial programs like the WHO, the United Nations International Children’s Emergency Fund, or the United Nations Population Fund can help extend this agenda.

The government can also make available the current mental health data reported by the mental health facility quarterly and provide an annual report based on those data on the state of mental health in the region. In this evidence-development process, key indicators are needed to monitor the mental health of the region’s population. They will allow for timely decision-making and adjustment when necessary. The product of these researches should then be disseminated to all the stakeholders so that their decisions can reflect the best available knowledge [2].

## Conclusion

The mental health system in the West Region of Cameroon still has many challenges and improvements that are required. However, the government is making efforts to fill this gap. One mental health nursing school was created in the region in the past two years; six out of the 20 districts have mental health services, and the first psychiatrist in the region arrived during our data collection. These efforts need to use an evidence-based and inclusive approach with a monitoring system for a better outcome.

## Limitations

In this study, the information provided by key informants may reflect their opinions more than the actual facts. The information collected from the records were altered by some missing information and the quality of the information reported. Some indicators such as the number of psychiatric beds available in the community-based psychiatric inpatient unit may not reflect the size of the service offered because most of the time, even though they did not have exclusive beds, they could admit a patient on any bed available in another unit. Some mental health units frequently have students that are there for internships and contribute to the service being offered but were not recorded in our study. Some other personnel were trained in HIV programs to offer psychological support to HIV patients, but they were not linked with the formal mental health system of the region and were not recorded in the study. This data reflected the situation in 2020, and things seem to be changing at the ground level in Cameroon.

## Data Availability

The data that supports the findings of this study are available from the corresponding author upon request.

## Abbreviations

WHO: World Health Organization
WHO-AIMS: World Health Organization Assessment Instrument for Mental Health Systems
LMIC: Lower- and Middle-income Countries
FOJCAM: Fondation Olympia Jujitsu Cameroun
